# A Noise Tolerant Spread Spectrum Sound-Based Local Positioning System for Operating a Quadcopter in a Greenhouse

**DOI:** 10.3390/s20071981

**Published:** 2020-04-01

**Authors:** Zichen Huang, Lok Wai Jacky Tsay, Tomoo Shiigi, Xunyue Zhao, Hiroaki Nakanishi, Tetsuhito Suzuki, Yuichi Ogawa, Naoshi Kondo

**Affiliations:** 1 Graduate School of Agriculture, Kyoto University, Kyoto 6068502, Japan; 2 Department of Ocean Mechanical Engineering, National Fisheries University, Shimonoseki 7596595, Japan; 3 Graduate School of Engineering, Kyoto University, Kyoto 6158540, Japan

**Keywords:** precision agriculture, spread spectrum sound, local positioning system, quadcopter, acoustic noise tolerance

## Abstract

Quadcopters are beginning to play an important role in precision agriculture. In order to localize and operate the quadcopter automatically in complex agricultural settings, such as a greenhouse, a robust positioning system is needed. In previous research, we developed a spread spectrum sound-based local positioning system (SSSLPS) with a 20 mm accuracy within a 30 × 30 m greenhouse area. In this research, a noise tolerant SSSLPS was developed and evaluated. First, the acoustic noise spectrum emitted by the quadcopter was documented, and then the noise tolerance properties of SSSounds were examined and tested. This was done in a greenhouse with a fixed quadcopter (9.75 N thrust) with the positioning system mounted on it. The recorded quadcopter noise had a broadband noise compared to the SSSound. Taking these SSSound properties into account, the noise tolerance of the SSSLPS was improved, achieving a positioning accuracy of 23.2 mm and 31.6 mm accuracy within 12 × 6 m for both Time-division Multiple Access (TDMA) and Frequency-division Multiple Access (FDMA) modulation. The results demonstrate that the SSSLPS is an accurate, robust positioning system that is noise tolerant and can used for quadcopter operation even within a small greenhouse.

## 1. Introduction

Drones are beginning to play an important role in precision agriculture, as they provide farmers with rapid and semi-autonomous data about the crops. The benefits of using drone implementation have been identified [1] as: reduced measurement and data collection time, precision of measured data, increased yields, decreased costs of fertilizers and pesticides, and so on. To date, most applications have focused on outdoor operations, some applications are already commercialized, such as monitoring the health of crops by using Normalized Differential Vegetation Index (NDVI) in open fields [2] and greenhouses [3], detecting [4] and mapping [5] weeds, surveying the agricultural field [6], and spraying pesticides and fertilizers [7]. Quadcopters, typically small drones with four motors, with less air turbulence have attracted the interest of researchers for measuring environmental variables [8] in a greenhouse, such as temperature, humidity, CO2 concentration, and so on. Flying quadcopters in greenhouses can also accomplish pollination and yield estimation [9]. However, for further automated quadcopter operations in greenhouses, a robust positioning system is a critical requirement.

Unfortunately, the ubiquitous Global Navigation Satellite System (GNSS) cannot be used as a positioning system in greenhouses due to large indoor positioning errors [10]. Indoor positioning is an important part of Internet of Things (IoT) and plays an important role in improving most services in IoT [11,12]. Moreover, many of the other commercialized indoor positioning systems use Ultra-Wide Band (UWB) signals that only have a 100 mm accuracy [13], and can interfere with wireless systems in Industrial Scientific Medical and Mobile cellular bands [14]. Other research has used a machine vision system for localizing the quadcopter with color markers on the motors [15]. Even this system only has a 128 mm positioning accuracy due to camera resolution and limited coverage, due to the field of view. On the other hand, sound-based positioning systems offer an attractive alternative with high accuracy and low cost.

Our research team has focused on developing a spread spectrum sound (SSSound) based positioning system. The potential advantages of such a sound-based positioning system include: high accuracy, low cost, obstacle tolerance, and no interference with electromagnetic waves [16]. To date, we have developed a SSSound-based local positioning system (SSSLPS) that can estimate position in a 30 × 30 m coverage area with an accuracy of 20 mm (average Root Mean Square Error (RMSE)) in the two-dimensional (2D) plane [17], and measure orientation on a robot with two receivers mounted on it to a 2.8° accuracy [18]. 

In previous research, the SSSLPS has used a passive localization structure, where the speaker is mounted on a ground robot, as well as a quadcopter [19]. In order to localize multi-robots in the greenhouse, a new positioning system architecture that sets the microphone [20] on the robot with either Time-division Multiple Access (TDMA) or Frequency-division Multiple Access (FDMA) is needed to remove the interference between speakers. Considering that the quadcopter emits a loud acoustics noise that may interfere with the SSSLPS, the noise tolerance of the SSSLPS under these conditions needs to be evaluated.

The objectives of this research are first, to document the acoustic noise emitted from a model quadcopter operated at various thrust levels and using different propellers. Then, with reference to the properties of this noise, improve the noise tolerance of the SSSound signals. Finally, the performance of this SSSLPS mounted on a quadcopter in a greenhouse was evaluated.

## 2. Spread Spectrum Sound-Based Local Positioning System

**Figure 1:**
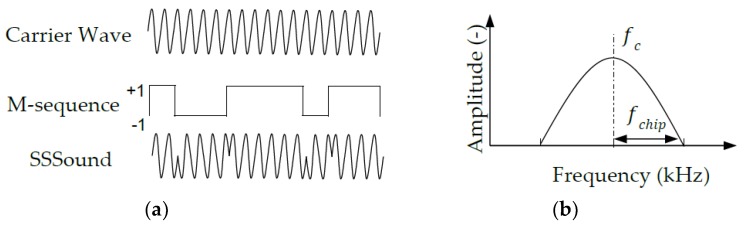
Generation (**a**) and frequency range (**b**) of Spread Spectrum Sound (SSSound).

Figure 1a illustrates the modulation of the SSSound. This example is typical of the SSSound used in our research. It uses a 1023 M-sequence length, 24 kHz carrier wave ($f_{c}$), and 12 kcps chip rate ($f_{chip}$). The SSSound is generated by a Binary Phase Shift Keying (BPSK) modulation method that multiplies the carrier wave with an M-sequence. The M-sequence [21] is a kind of pseudo-noise sequence with a single peak of auto correlation [22]. Both the carrier wave frequency and chip rate determine the frequency range of the SSSound (Figure 1b).

The generation of SSSound s(*n*) can be described by the following equation:

(1) $$s\left(n\right)=\sin\left(\frac{2\pi f_{c}n}{f_{s}}\right)\times M\left(\mathrm{floor}\left(\frac{f_{chip}}{f_{s}}\times n\right)\right),$$

where $f_{c}$. (Hz) is the frequency of the carrier wave, $f_{chip}$. (cps: chip per second) is the chip rate, $f_{s}$ (Hz) is the sampling frequency, *n* = 0, 1, 2 … *k*−1 (*k* is the length of SSSound), M is the M-sequence, and floor(*x*) is floor function.

The system emits SSSound and the trigger signal simultaneously, and the sound signal is then received by the microphone. After N samples are recorded, the cross-correlation value is calculated as follows:

(2) $$\begin{array}{c} C\left(t\right)=\sum_{n=0}^{N-1}s\left(n\right)r\left(n+t\right)\\ C_{Normalized}\left(t\right)=C\left(t\right)/C_{max} \end{array}$$

where *t* is the time of received data, r(t) is the received signal, $C_{max}$ is the maximum cross-correlation value C(t), and $C_{Normalized}\left(t\right)$ is the normalized cross-correlation value. $C_{Normalized}\left(t\right)$ is used for peak detection (see below).

**Figure 2:**
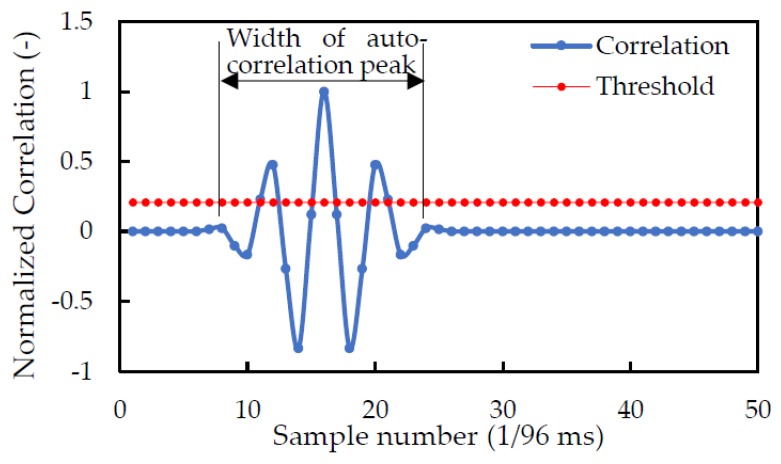
Auto-correlation value.

Figure 2 shows an example of an auto-correlation value with an obvious peak, calculated by Equation (2) assigned the same received signal and SSSound signal. The SSSound properties in this example are a sampling frequency ($f_{s}$) of 96 kHz, a chip length of eight samples (chip rate is 12 kcps), and a carrier wavelength of four samples (carrier frequency is 24 kHz). The width of the auto-correlation peak of the M sequence is 16 samples (twice the chip length), therefore the width of the auto-correlation peak of the SSSound is also 16 samples. There are three observed peaks over the width of the auto-correlation peak, because of the carrier wave effect. The number of samples between the peaks is four samples (carrier wavelength). The center peak represents the arrival time of the SSSound signal. The red line is the threshold value calculated by the following function, which is used to detect the highest of the peaks in the region:

(3) $$c_{th}=C_{ave}+4\sigma_{corr}$$

where, $C_{ave}$ and $\sigma_{corr}$ are the average absolute value and standard deviation of normalized cross-correlation, respectively. In this example, the time of the second peak over the threshold value was used as the received time of SSSound, $t_{s}$ (s).

The sound velocity, $v_{j}$ (m s^−1^), and distance, $d_{j}$ (m), from the speaker, j, to the microphone can be calculated by the following equations:

(4) $$v_{j}=331.5+0.61\times T_{j}$$

(5) $$d_{j}=v_{j}\left(t_{s}-t_{t}-t_{delay}\right)$$

where, $T_{j}$ (°C) is the average temperature between microphone and speaker, *j*; $t_{t}$ (s) is received time of trigger signal; and $t_{delay}$ (s) is the time delay offset caused by the High Pass Filter (HPF) circuit of the speaker.

**Figure 3:**
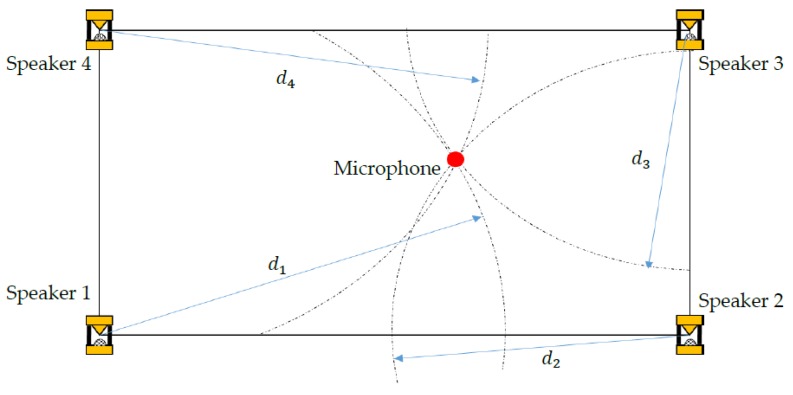
Basic setup of Spread Spectrum Sound-based Local Positioning System (SSSLPS).

The basic setup of the SSSLPS (Figure 3) consists of four speakers at the corner and one microphone on the robot with a Time of Arrival (TOA) – based algorithm [23]. The radius of the four circles is the distance between the speaker and the microphone (red point in Figure 3). The 3D position can be estimated using the least-squares algorithms [24].

## 3. Materials and Methods

### 3.1. Acoustic Noise Measurement

**Figure 4:**
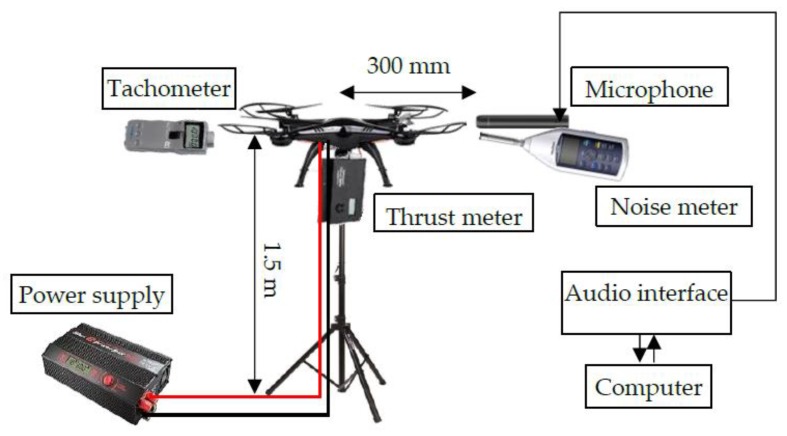
Setup for measuring the noise spectrum.

The setup for measuring the noise spectrum is shown in Figure 4. The quadcopter model consists of an Electronic speed control (ESC, 50A, KYWALKER), brushless motors (FC4250-6T KV720, FSD), and power supply (HRC44174, HiTEC). Three kinds of carbon propellers, models 8 × 3.8, 10 × 4.5, and 12 × 4.5 (by GEMFAN), with diameters of 20.5 cm, 25.5 cm, and 30.5 cm, respectively, were used in the experiments. To document the acoustic noise emitted from the quadcopter, three different propellers were used in the experiment. The Arduino (UNO REV3, Arduino) was used to control the ESC using Pulse Width Modulation (PWM) signals. The quadcopter was fixed on a stand with a thrust meter (Tahmazo, OK Model, accuracy 5 g). A tachometer (HT-5100 Ono Sokki, accuracy ±1 r/min) and a reflective board attached on the motor were used to measure the Revolutions Per Minute (RPM) of the motor. The height of the quadcopter and the microphone (ECM-100N, Sony, frequency response ±3 dB) were set at 1.5 m above the ground.

The SSSound signal covers the ultrasonic range, so a noise meter (LA-4440, Ono Sokki, frequency response ±1 dB) and microphone were used to evaluate the frequency spectrum of the acoustic noise emitted from the quadcopter. The microphone was connected to the computer by an audio interface (OCTA-CAPTURE UA-1010, Roland) with a constant gain value. 

The relationship between the sound pressure level (dB) measured by the noise meter and the signal recorded by microphone can be expressed by the following equation:

(6) $$\begin{array}{c} L_{noise\;meter}=10\;\log_{10}\frac{{p_{m}}^{2}}{p_{0}^{2}}\\ p_{m}=\sqrt{\frac{\sum {\left(Gain\times signal\right)}^{2}}{N}}\\ Gain=\sqrt{\frac{p_{0}^{2}\times N}{\sum {\left(signal\right)}^{2}}\times 10^{\frac{L_{noise\;meter}}{10}}} \end{array}$$

where, $L_{noise\;meter}$ is the sound pressure level measured by the noise meter, $p_{m}$ is the sound pressure measured by the microphone, $p_{0}$ is the reference sound pressure ($2\times 10^{-5}\;\mathrm{Pa}$), Gain is the system gain, and signal is the received signal.

**Figure A1:**
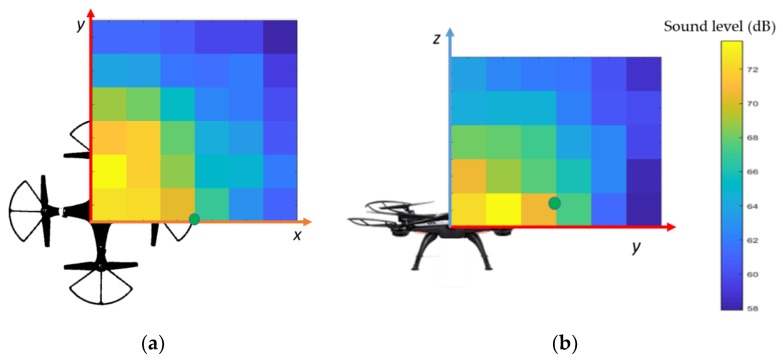
The horizontal (**a**) and vertical (**b**) noise distribution over the quadcopter.

The noise meter measures sound pressure level from 10 to 20 kHz, so the Gain in Equation (6) can be calculated using the received signal with the same frequency range as the noise meter. With a 96 kHz sampling frequency and a Band Pass Filter (BPF) for the received signal, the sound pressure level can be measured over the above frequency range. The microphone was set at 300 mm from the center of the quadcopter (Figure A1). The thrust of the quadcopter was set to 4.91 N, 9.73 N, and 14.72 N (±0.45 N). A 5 s sound signal was recorded at each condition under the stable thrust.

### 3.2. Noise Tolerance Against Quadcopter

Three groups of SSSound signals were generated, taking into consideration that the period of the M-sequence(*M_length_*), the frequency of the carrier wave, and chip rate can affect the noise tolerance of the SSSound. The properties of these signals are shown in Table 1.

The strength of SSSound, in respect to the noise, is described as Signal to Noise ratio of correlation (*SNR_corr_*) [19] by the following equation:

(7) $$SNR_{corr}=\frac{C_{peak}}{C_{absave}}$$

where, $C_{peak}$ is the correlation value at the received time of the SSSound. $C_{absave}$ is the average absolute correlation value, except in the 48 samples peak region, which is the maximum peak width of the auto-correlation. The larger the mean *SNR_corr_* value, the larger the noise tolerance. Normally, the arrival time of the sound signal cannot be detected when the *SNR_corr_* value is smaller than 7.

In addition to the experimental setup shown in Figure 4, a speaker (FT28D, Fostex Company), connected to the audio interface by an amplifier (Kama Bay Amp Rev. B, Scythe Inc.), was setup to emit the SSSound. The 1 kHz HPF was connected to the speaker. The distance between the speaker and the microphone was set at 1 m, at the same height of 1.5 m. The sound pressure level of the emitted SSSound was adjusted to 95 dB, measured 100 mm from the speaker using white noise. The thrust of the quadcopter was set at 9.73 ± 0.68 N. With the operating quadcopter fixed on the stand, the $SNR_{corr}$ of each signal was measured 50 times for the three types of propellers.

### 3.3. Positioning Experiment in the Greenhouse with Quadcopter Noise

**Figure 5:**
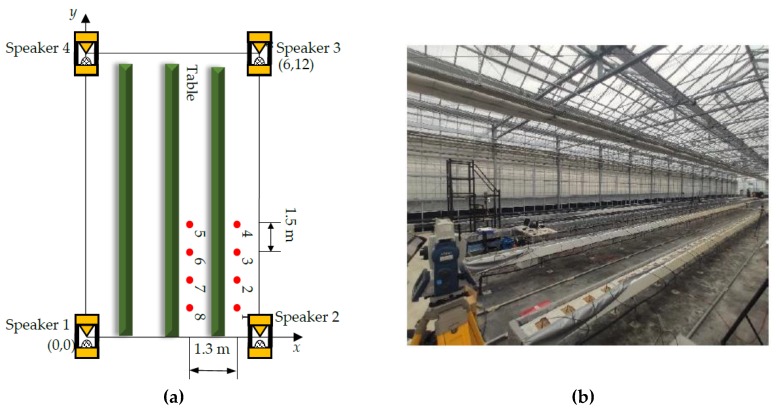
Experimental setup (**a**) and view (**b**) in the greenhouse.

To evaluate the effect on the SSSLPS of the quadcopter emitted noise, an experiment was conducted in an affiliated greenhouse of Kyoto University in Kizugawa city. The coverage area of the experiment was set as 12 × 6 m, given the possible limited range of SSSound in the presence of quadcopter noise. Figure 5 shows the setup in the greenhouse. There were empty raised rockwool beds on bare soil in the greenhouse. The four speakers were set at each of the corners, and the quadcopter was sequentially set at each red point shown in Figure 5. The microphone was attached 30 cm from the center of the quadcopter. The speakers and microphone height were set at 150 cm. A commercial omnidirectional microphone (SPM0404UD5, Knowles Electronics) was used in this experiment. The temperature of the speakers and microphone were monitored using wireless thermometers (3670 Hioki, accuracy 0.1 °C) in order to estimate sound velocity for distance measurements. The temperature during this experiment ranged from 26.4 to 37.3 °C. This research focuses on noise tolerance in the presence of quadcopter acoustic noise, so the quadcopter was fixed on a stand (Figure 4). The offset, $t_{delay}$, was calibrated as 0.036 ms for the four speakers. The position of the microphone, $P_{j}$, was measured 50 times at each red point in Figure 5 by the SSSound system, with the quadcopter running at a thrust of 9.75 ± 0.58 N using propeller model 10 × 4.5. The positioning accuracy of the SSSLPS was evaluated in comparison to the position, $P_{ts}$, measured by the total station (SRX5XT32T-11, Sokkia, accuracy 1.5 + 2ppm× measurement distance mm). The RMSE of position, $Positioning_{RMSE}$, at each position is calculated by the following equation:

(8) $$Positioning_{RMSE}=\sqrt{\frac{1}{50}\overset{50}{\underset{j=1}{\sum }}{\left(P_{j}-P_{ts}\right)}^{2}}$$

**Figure 6:**
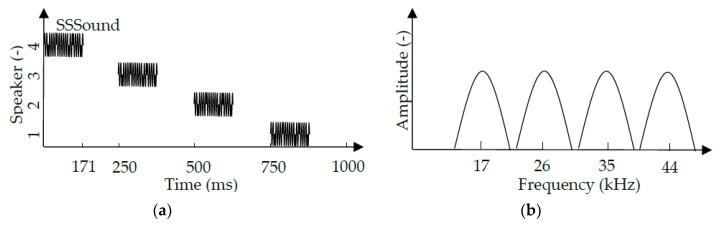
Time-division Multiple Access (TDMA) (**a**) and Frequency-division Multiple Access (FDMA) (**b**) signal.

Both TDMA and FDMA signals were evaluated in the experiment. The TDMA signal used was based on the SSSound signal generated by a 2047 length M-sequence, 24 kHz carrier wave, and 12 kcps chip rate. The length of the SSSound was 171 ms. Channels 1 to 4 were the SSSound channels of the speakers, as shown in Figure 6a. The interval between the speakers was set at 0.25 s, so one cycle of the TDMA signal was 1 s. The frequency-domain of the FDMA signal is shown in Figure 6b. The four speakers produced 17, 26, 35, and 44 kHz carrier waves in the corresponding channels with a 4 kcps chip rate and a 1023 length M-sequence. The length of each SSSound signal in the FDMA channel was 256 ms, which is much longer than the 171 ms length signal in the TDMA channel. The received signal, *N*, of TDMA and FDMA was sampled 24,000 and 48,000 times for each channel, respectively. At each measured position, the TDMA and FDMA based SSSound was measured with and without the operating quadcopter to compare the effect of quadcopter emitted noise.

## 4. Results and Discussions

### 4.1. Acoustic Noise of the Quadcopter

**Figure 7:**
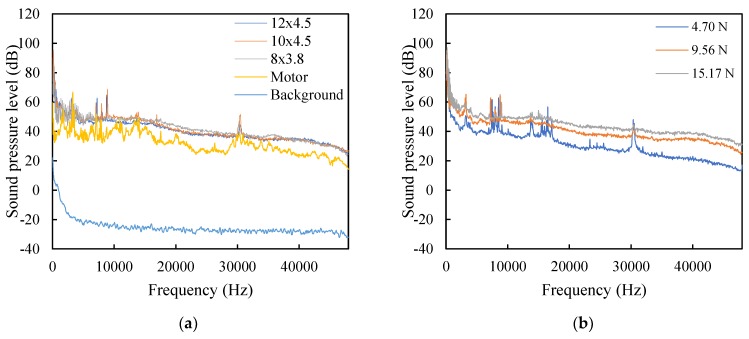
Acoustic noise spectrum of the quadcopter noise at 9.73 N thrust (**a**) and spectrum change with thrust using propeller model 10 × 4.5 (**b**).

Figure 7a shows the frequency spectrum of the acoustic noise emitted from the quadcopter with three different propeller sizes, the noise is from the motors at 4013 RPM and background noise. The RPM of the models 80 × 3.8, 11 × 4.7, and 12 × 4.5 were 3524, 4210, and 8356 RPM, respectively. Based on previous acoustic noise classification from quadcopters [25], the acoustic noise in this experiment was classified into low frequency range (0–10 kHz) and high frequency range (>10 kHz). In the low frequency range, shaft rate, blade passing frequency, rotor self-noise, and their harmonics could be observed.

The difference of the noise spectrum with the three propellers model mainly exists in the low frequency because of the different motor RPM. Other research [26] has pointed out that acoustic noise from agricultural machinery, such as mower engines, hand movers, and combine harvesters, are generally in the low frequency range. Thus, to avoid noise interference from the quadcopter or other agricultural machineries, the SSSound signal should be in a higher frequency range. In this research, the minimal SSSound frequency was set as 10 kHz. Acoustic noise in high frequencies above 10 kHz is described as broadband noise, which causes turbulent flow over the blades; something that was also observed in previous research [25,27].

Noise around 30.5 kHz is presumably from laminar boundary layer vortex shedding [25]. This noise is coupled to acoustically excited feedback loops, generated between the trailing edge and instability waves upstream of the trailing edge [28]. The noise spectrum changed with thrust when using the 11 × 4.7 propeller, as shown in Figure 7b. As the thrust increased from 4.70 to 15.17 N, broadband noise at higher frequencies increased. Thus, the noise generated by the quadcopter was considered to be broadband noise, compared to that of the SSSound signal used in this research.

### 4.2. Noise Tolerance Against Quadcopter

**Figure 8:**
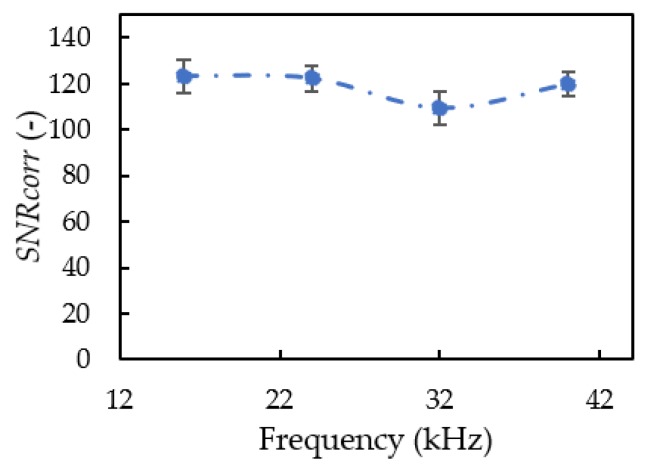
The effect of carrier wave changes to the noise tolerance.

Figure 8 shows changes in noise tolerance with the carrier frequency. The *SNR_corr_* value fluctuated around 13.9 with the four tested carrier frequencies. The coverage of these signals was 8 to 44 kHz. This frequency range includes vortex shedding around 30.5 kHz, so the *SNR_corr_* value at 28 kHz was slightly lower, around 9.3, than the *SNR_corr_* value at other carrier frequencies. The standard deviation was around 7, associated with the three different propeller types tested. The average *SNR_corr_* value of the four carrier waves was 118.7. Based on the noise spectrum emitted from the quadcopter, these carrier frequencies can be affected by noise at 30.5 kHz, and its effect on the *SNR_corr_* value was around 7.8% (=9.3/118.7 × 100%).

**Figure 9:**
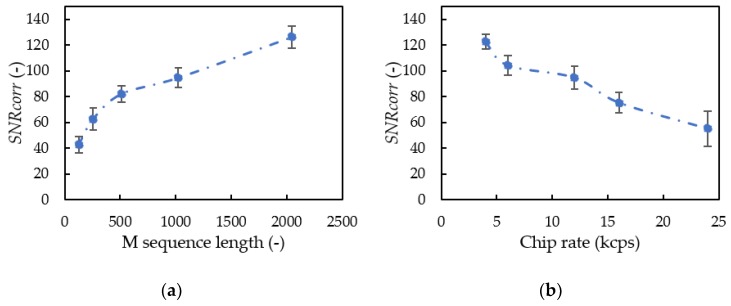
Noise tolerance change with M sequence length (**a**) and chip rate (**b**).

The longer M-sequence lengths and shorter chip rates trialed had larger *SNR_corr_* values (Figure 9). By doubling the length of the M-sequence, the *SNR_corr_* value increased by about 20. The M-sequence is multiplied by the sine wave (Equation (1)), so the correlation value increases as the M-sequence length increases. As the chip rate increased, the noise tolerance decreased. A chip rate of 24 kcps had the largest decrease in noise tolerance, because the 24 kHz carrier wave and 24 kcps chip rate compose the SSSound signal with 48 kHz broadband frequency that covered the quadcopter noise in the low frequency.

The length of the SSSound signal ($L_{S}$, unit s) can be calculated by the following equation:

(9) $$L_{S}=\frac{M_{length}}{f_{chip}}$$

**Figure 10:**
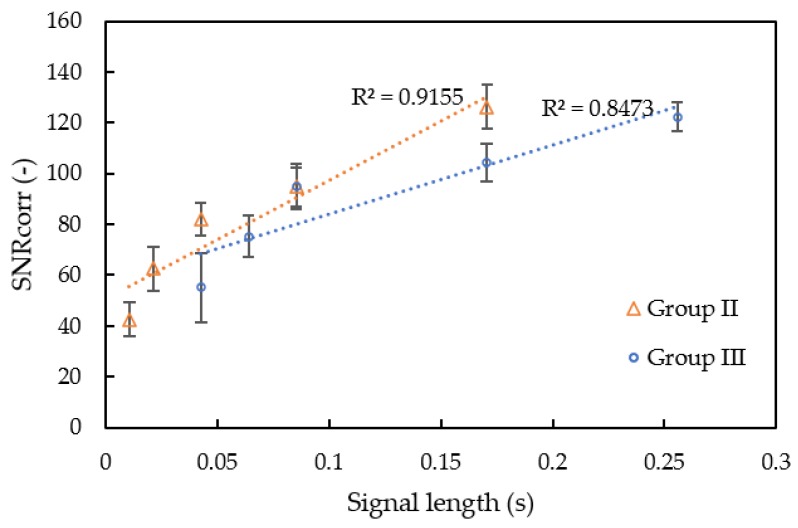
Signal length with the noise tolerance.

A smaller chip rate and longer M sequence length would have better noise tolerance. Figure 10 shows the correlation coefficient between signal length and the *SNRcorr* value. The correlation coefficient, R^2^, between signal length and *SNRcorr* value was larger than 0.84 for both M sequence length and chip rate. A shorter chip rate means more sine wave was coded into the M sequence, while the sine wave itself didn’t contribute much to noise tolerance, which is why the ultrasound with single frequency has low noise tolerance. The slope in Figure 10 of the M sequence length is larger than that for chip rate, so increasing the M sequence length is an effective means to increase noise tolerance of the SSSound system.

### 4.3. Positioning Results in Greenhouse with Quadcopter Noise

**Figure 11:**
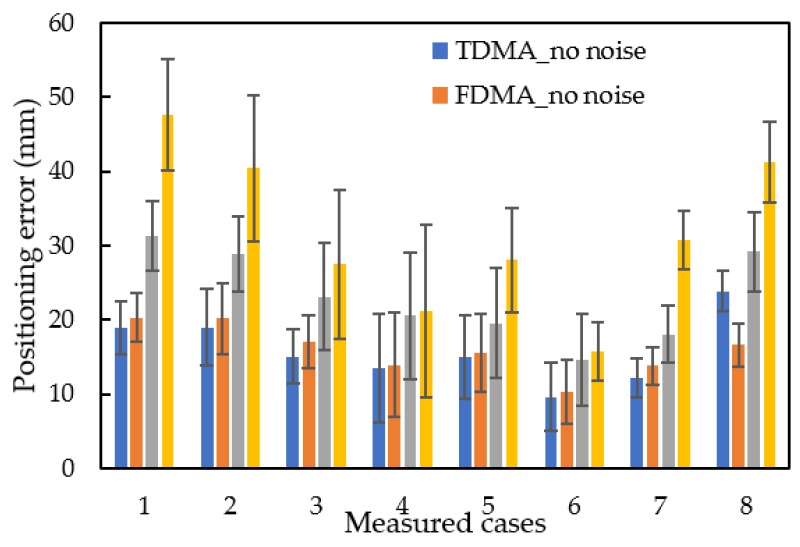
Positioning error.

The RMSE of the positioning error for both TDMA and FDMA signals when the quadcopter was operating and without quadcopter noise is shown in Figure 11. The RMSE of the 2D positioning of the TDMA modulation with and without quadcopter noise, was 15.9 mm and 23.2 mm, respectively. The RMSE of FDMA modulation with and without noise, was 16.0 mm and 31.6 mm, respectively. The error bars are the standard deviation of 50 measurements of each signal. Positions 1, 2, and 8 have errors that were larger than for the other positions; presumably, because these three positions were around 45 mm distance error from speaker 4 to the microphone.

**Figure 12:**
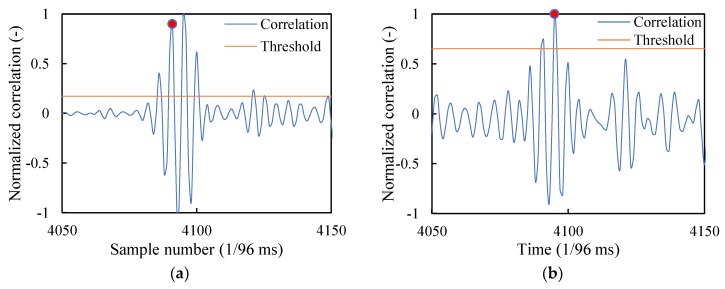

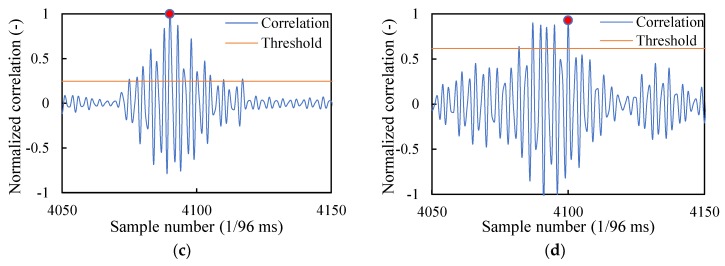
Correlation of the TDMA (**a**,**b**) and FDMA (**c**,**d**) signals with the non-operating quadcopter (**a**,**c**) and operating quadcopter (**b**,**d**).

In the presence of quadcopter noise, the positioning errors and error bars for both the TDMA and FDMA modulations increased. Figure 12 shows the correlation between the TDMA and FDMA signals at position 1, from speaker 4 to the microphone. The red points in Figure 12 indicate the detected peak using the threshold method. The width of the peak region for the TDMA and FDMA signals were 16 and 48 sample numbers. Figure 12a is the correlation for the TDMA signal with no quadcopter noise. The second peak over the threshold was the arrival time of the SSSound signal. With the interference of the quadcopter noise, the correlation peak changes from Figure 12a to Figure 12b and the wrong peak was detected, so the distance measurement error increased and lead to the positioning error increase. The average absolute correlation and standard deviation of the correlation increased, so the threshold in Figure 12b is larger than the threshold without the quadcopter noise (Figure 12a). At this position, the positioning error of SSSLPS using TDMA signal were 18.9 mm without the quadcopter noise, and 31.3 mm with the quadcopter noise. For the FDMA signals with (Figure 12d) and without (Figure 12c) the quadcopter noise, the larger distance measurement error appears because the FDMA has a greater width of the peak region, and the correct peak is difficult to be detected. The positioning accuracy can be affected by sampling frequency [29], the error of correlation peak, and the error of sound velocity measurement [30].Without the quadcopter noise, the correct correlation peak of both TDMA and FDMA signals can be detected. In this experiment, the error of sound velocity estimation (Equation (4)) contributes to the positioning error without the quadcopter noise. The correct peak is difficult to be detected when there was interference with the quadcopter noise.

**Figure 13:**
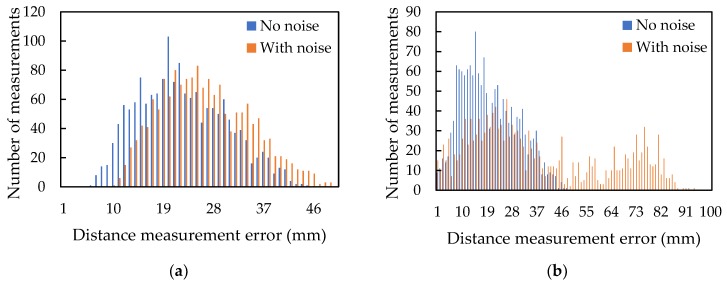
Histogram of the absolute distance measurement error of TDMA (**a**) and FDMA (**b**) signals.

Figure 13 shows the histogram of the absolute distance measurement error of TDMA (Figure 13a) and FDMA (Figure 13b) signals without the quadcopter noise (blue legend) and with the quadcopter noise (orange legend). The mean absolute error of distance measurement of the TDMA modulation without and with quadcopter noise, was 22.5 mm and 26.4 mm, respectively. With the quadcopter noise, the mean absolute distance error using FDMA signals increased from 19.8 mm to 37.2 mm because of the wrong peak detection. Compared with the TDMA signals, the FDMA signals have larger distance measurement error when the quadcopter noise exists. So, the TDMA signal has better accuracy of positioning and distance measurement with the quadcopter noise, due to smaller peak detection error than in FDMA signals.

**Figure 14:**
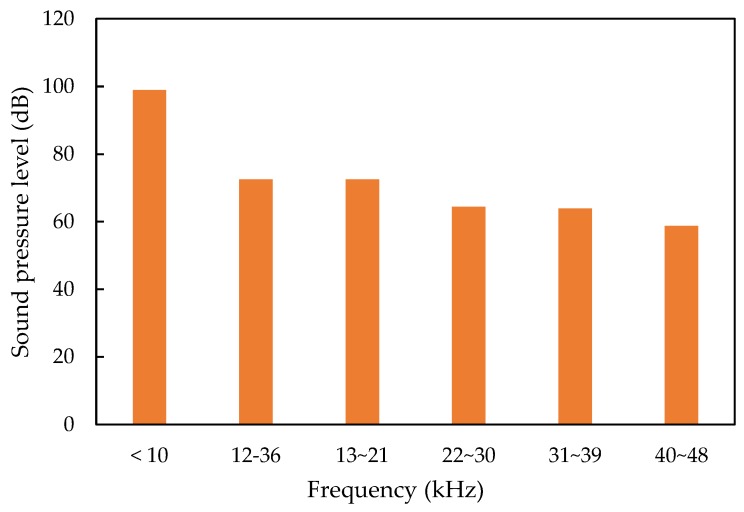
The sound pressure level of the TDMA and FDMA channels.

The sound pressure level of the TDMA and FDMA channels using three propeller models are shown in Figure 14. The sound pressure level of the noise in TDMA 12 kHz to 36 kHz is around 72 dB. The sound pressure level of the four channels in FDMA are 73, 64, 64, and 59 dB, which decreased because the noise spectrum in high frequencies decreased gradually (Figure 6).

**Figure 15:**
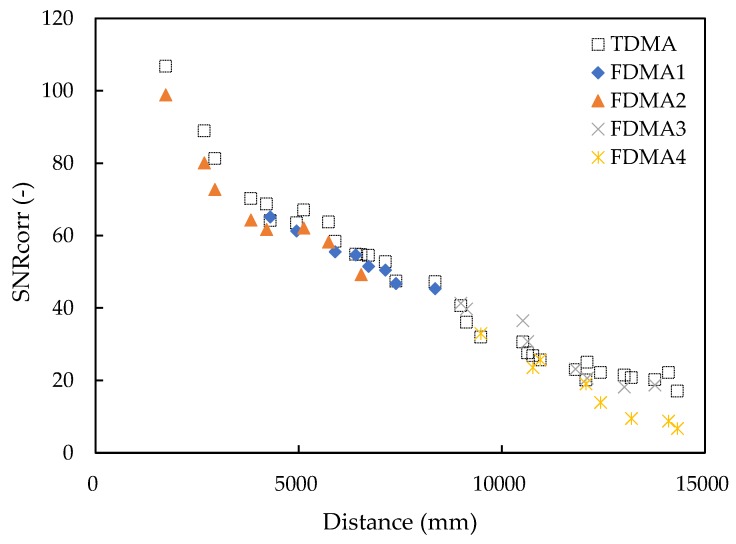
Signal strength decrease with distance.

Figure 15 shows that the *SNR_corr_* value of the TDMA signal and four channels of the FDMA signal decreased with the distance from the acoustic noise by the quadcopter. The four channels of FDMA were labeled as FDMA1 to FDMA4. The TDMA and FDMA have the same decreasing trend with distance, because the noise at the microphone is relatively constant and the sound pressure level of the SSSound at the microphone is decreased. The frequency in channel 4 of the FDMA is 40 kHz to 48 kHz. The sound pressure level attenuation in high frequencies are stronger than low frequencies [31,32], so the noise tolerance of channel 4 of FDMA decreased faster than other channels. The distances from FDMA channel 4 that were larger than 14 m were difficult to detect. While the *SNR_corr_* value of the TDMA signal at 14327.1 mm is 17.1, the TDMA signal can be used for a longer distance measurement. For the FDMA signals, due to the sound pressure level damping in high frequencies, the measurement distance is limited in 15 m when the thrust of the quadcopter reaches 9.75 N. Considering the coverage distance, the TDMA based SSSLPS has a larger potential than the FDMA system to be used for quadcopters in greenhouses.

## 5. Conclusions

To evaluate the ability of the SSSLPS to be used as a positioning system on a quadcopter, the acoustic noise spectrum from the quadcopter and noise tolerance of the SSSLPS were analyzed. Then, the experiment in the greenhouse was conducted to evaluate the accuracy of SSSLPS on the quadcopter. The quadcopter noise in the SSSound range is mainly broadband aerodynamic noise. To improve the noise tolerance of SSSLPS, the SSSound signal can use longer M-sequence lengths and shorter chip rates. The longer M-sequence lengths can be used for the TDMA system. The shorter chip rates can be used for the FDMA system. The positioning error of the FDMA system is 31.6 mm, which is larger than the 23.2 mm positioning error of the TDMA system with quadcopter noise. This is because the FDMA signals with the small chip rate were affected by the carrier wave, and the correct peak was difficult to detect. Meanwhile, considering the sound pressure level damping in the high frequencies of the FDMA system, the TDMA is better used for the quadcopter, since it has larger measurement areas than the FDMA system.

The moving quadcopter, with the TDMA modulation system, has a larger error during movement since the four speakers emit sound signals at different times and need high positioning update frequency. The Inertial navigation system uses the Inertial Measurement Unit (IMU) and can be used to estimate the position [33], but with accumulated error. One possible solution is sensor fusion with the IMU and SSSLPS, using Extended Kalman Filter (EKF) to minimize the positioning error. More work needs to be done to enlarge the coverage of this system. In this experiment, we measured the position in a 12 × 6 m area with four speakers. To enlarge the coverage area of SSSLPS, the typical method is to use more nodes [34,35] and more speakers in our system.

## Figures and Tables

**Table 1:** SSSound pairs for comparison.

Group	*M_length_*	$f_{c}\;(\mathrm{kHz})$	$f_{chip}\;(\mathrm{kcps})$
Ⅰ	1023	20, 24, 28, 32	4
Ⅱ	127, 255, 511, 1023, 2047	24	12
Ⅲ	1023	24	4, 6, 12, 16, 24

## Appendix A. Acoustic Noise Distribution over The Quadcopter

Figure A1 is the horizontal (Figure A1a) and vertical (Figure A1b) acoustic noise distribution over the quadcopter in the TDMA frequency range within the 10 × 10 cm grid. The setting used 9.46 ± 0.57 N thrust and propeller model 10 × 4.7. Based on this distribution, the microphone was set at 300 mm from the center of the quadcopter and 8 cm a the quadcopter.
